# Influence of the body positions adopted for resistance training on intraocular pressure: a comparison between the supine and seated positions

**DOI:** 10.1007/s00417-023-06009-0

**Published:** 2023-02-16

**Authors:** Paula M. Lara, Beatriz Redondo, Daniel Jerez-Mayorga, Dario Martínez-García, Amador García-Ramos, Jesús Vera

**Affiliations:** 1 CLARO (Clinical and Laboratory Applications of Research in Optometry) Laboratory, Department of Optics, Faculty of Sciences, University of Granada, Campus de La Fuentenueva 2, 18001 Granada, Spain; 2 Department of Physical Education and Sport, Faculty of Sport Sciences, University of Granada, Granada, Spain; 3 Strength & Conditioning Laboratory, CTS-642 Research Group, Department of Physical Education and Sport, Faculty of Sport Sciences, University of Granada, Granada, Spain; 4 Exercise and Rehabilitation Sciences Institute, School of Physical Therapy, Faculty of Rehabilitation Sciences, Universidad Andres Bello, 7591538 Santiago, Chile; 5 Department of Sports Sciences and Physical Conditioning, Faculty of Education, Universidad Católica de La Santísima Concepción, Concepción, Chile

**Keywords:** Exercise, Physical activity, Eye health, Glaucoma management, Rebound tonometry

## Abstract

**Objectives:**

A variety of factors are known to mediate on the intraocular pressure (IOP) response to resistance training. However, the influence of the body position adopted during resistance training on IOP remain unknown. The objective of this study was to determine the IOP response to the bench press exercise at three levels of intensity when performed in supine and seated positions.

**Methods:**

Twenty-three physically active healthy young adults (10 men and 13 women) performed 6 sets of 10 repetitions against the 10-RM (repetition maximum) load during the bench press exercise against three levels of intensity (high intensity: 10-RM load; medium intensity: 50% of the 10-RM load; and control: no external load) and while adopting two different body positions (supine and seated). A rebound tonometer was employed to measure IOP in baseline conditions (after 60 s in the corresponding body position), after each of the 10 repetitions, and after 10 s of recovery.

**Results:**

The body position adopted during the execution of the bench press exercise significantly affected the changes in IOP (*p* < 0.001, *η*_p_^2^ = 0.83), with the seated position providing lower increases in IOP levels compared to the supine position. There was an association between IOP and exercise intensity, with greater IOP values in the more physically demanding conditions (*p* < 0.001, *η*_p_^2^ = 0.80).

**Conclusions:**

The use of seated positions, instead of supine positions, for the execution of resistance training should be prioritized for maintaining more stable IOP levels. This set of findings incorporates novel insights into the mediating factors on the IOP response to resistance training. In future studies, the inclusion of glaucoma patients would allow to assess the generalizability of these findings.

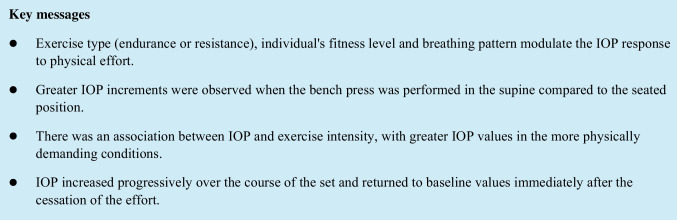

## Introduction

The health benefits of physical exercise practice are irrefutable, including risk reductions of at least 20–30% for more than 25 chronic medical conditions and premature mortality [1, 2]. Nevertheless, generic physical activity guidelines should not be used when working with clinical populations. Exercise prescription should be individualized based on underlying medical condition and functional status for optimal patient care [3].

Glaucoma is the leading cause of irreversible blindness worldwide [4]. As a result, public health strategies are being discussed to minimize the negative consequences of glaucoma, although the possibility to adopt different public health strategies vary significantly across countries [5]. Remarkably, the reduction and stabilization of intraocular pressure (IOP) are the only strategy that has proven to be beneficial for the prevention and management of glaucoma, with all the possible interventions (i.e., surgery, pharmacological treatment, or lifestyle) aiming to reduce and stabilize IOP levels [6–9]. In this regard, the effects of a lifestyle factor such as the physical exercise on IOP control have been extensively explored in recent years, with different types of physical exercise showing either a positive (i.e., IOP reduction) or negative (i.e., IOP increase) effect for the prevention and management of glaucoma [10].

Endurance-type exercises (e.g., running or cycling) performed at low to moderate intensities cause an IOP reduction [11, 12], whereas resistance-type exercises (e.g., weightlifting) provoke an acute IOP rise which is accentuated at higher training intensities (i.e., using heavier loads and performing repetitions closer to muscular failure) [13–15]. In non-exercise contexts, there is scientific evidence that IOP values are greater in when adopting a horizontal position compared to the seated and upright positions [16–20]. Specifically, Vera and colleagues [21] found that the IOP response to reading at a close distance (30 cm) is dependent on body position, observing greater IOP values in the supine compared to a seated position. Remarkably, a number of resistance training exercises (e.g., bench press) can be performed while adopting different body positions (e.g., standing, sitting, lying down), but the differences in the IOP behavior when the same exercise is performed adopting different positions have never been examined.

In order to address the gaps found in scientific literature, we designed an experimental study to compare the impact of performing ten repetitions of the bench press exercise in supine and seated positions on IOP levels. Complementarily, these possible differences were determined for three exercise intensities, namely a control condition (no physical effort), medium-intensity condition (half of the 10-repetition maximum (RM) load), and high-intensity condition (10-RM load). Based on the accumulated scientific evidence in non-physical activities [16–21], we hypothesized greater IOP values when executing the bench press in a supine compared to a seated position. We also hypothesized that the IOP values will be higher for the more physically demanding exercise conditions [13, 14] and there will be a cumulative and progressive IOP rise as a function of accumulated physical effort [22].

## Methods

### Participants

An a priori sample size calculation was performed using the GPower 3.1 software. This software has been widely used in research studies and supports sample size and power calculation for various statistical methods (*F*, *t*, *χ*^2^, z, and exact tests) [23, 24]. For this analysis, we assumed an effect size of 0.20, alpha of 0.05, and power of 0.90, which projected a necessary sample size of 23 participants. At this point, 23 physically active individuals (10 men and 13 women) were recruited to participate in this study (age = 24.7 ± 4.8 years, body mass = 71.2 ± 12.4 kg and body height = 1.73 ± 0.09 m; data presented as mean ± SD). All participants were of Caucasian ethnicity and non-smokers. They were free of any systemic or ocular disease, were not taking any medication, and had no family history of glaucoma. The study followed the guidelines of the World Medical Association (Declaration of Helsinki) and was approved by the Institutional Review Board. Written informed consent was obtained from all participants before the initiation of the study.

### Intraocular pressure assessment

A rebound tonometer (Icare IC200, TiolatOy, INC. Helsinki, Finland) was used to measure IOP from the right eye. This new model has shown an acceptable level of reproducibility in the sitting and supine positions and a high agreement with Goldman applanation tonometry [25, 26]. The IC200 tonometer permits to accurately measure IOP when the subject is sitting, standing, or lying in a supine position due to its free angle measurement addition, and incorporates a light signal to minimize position-related errors. Also, this handheld tonometer has several advantages in practical terms (e.g., easy and rapid measurement, well-tolerated procedure, and does not require topical anesthesia or fluorescein), which makes this device a highly valuable tool for measuring IOP in applied settings. Following the manufacturer recommendation, IOP measurements were taken with the probe placed at a distance of 5 to 8 mm from the central cornea while the subject fixated on a distant target. IOP measurement was conducted by the same examiner (PL).

### Testing procedures

The first session was used to determine in a randomized order the 10-RM load (i.e., the load with which participants can perform a maximum of 10 repetitions) during the bench press exercise performed in both supine and seated positions. The session began with a warm-up consisting of jogging and upper-body dynamic stretching exercises. Subsequently, participants performed the first set against the 50% of their self-perceived 10-RM load. Participants were told to stop the set after 5 repetitions when they or an experienced strength and conditioning researcher identified that more than 10 repetitions could be performed. The load was then incremented in agreement between the participant and the researcher and a new set was performed after 3 min of passive rest. Participants needed between 3 and 6 sets to reach their 10-RM load. The 10-RM load was 39.04 ± 18.05 kg in the supine position and 53.57 ± 21.31 kg in the seated position.

The second session allowed us to test our hypotheses and consisted of 6 sets of the bench press exercise (2 positions (supine and seated) and 3 exercise intensities (control, medium intensity, and high intensity)). The 10-RM load and the 50% of the 10-RM load were used for the high- and medium-intensity conditions, respectively. In the control condition, participants simulated the same movement without using any additional load. The order of the sets was randomized and they were separated by 10 min of passive rest. Participants were asked to adopt the corresponding body position, and the baseline measurement was taken after maintaining this position for 60 s. During the exercise set, IOP was assessed immediately after performing each repetition (a total of 10 measurements) in a relaxed position (elbows fully extended). Lastly, IOP was measured after 10 s of passive rest (recovery measurement).

**Fig. 1:**
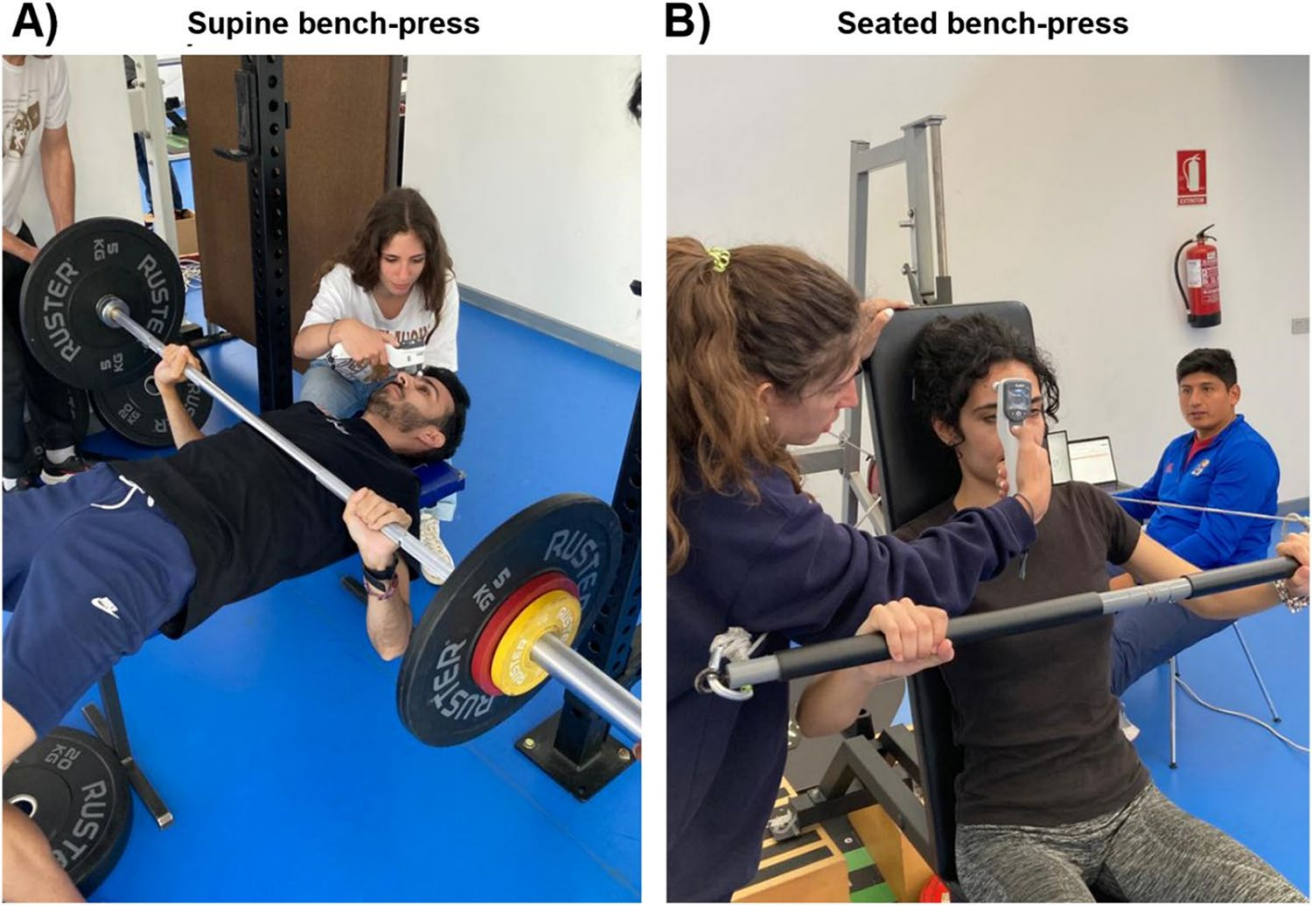
Photographs of the data collection. Panels **A** and **B** show the supine and seated bench press conditions, respectively

The supine bench press was performed with an Olympic free-weight barbell and weight disks (Fig. 1 panel A). The seated bench press was performed using a functional electromechanical dynamometer (FEMD) (Fig. 1 panel B) [27]. This FEMD (Dynasystem, Model Research, Granada, Spain) allows to perform a wide variety of movements and it can deliver a wide variety of stimuli (i.e., isokinetic, isotonic, elastic, isometric, inertial, eccentric, and vibration). The control core of this device permits to precisely regulate both force and angular velocity through a 2000-W electric motor. The user applies forces on a rope that winds on a roller, thus controlling and measuring both force and linear velocity. A load cell detects the tension applied to the rope and the resulting signal goes to an analog-to-digital converter with 12-bit resolution. Displacement and speed data are collected with a 2500-ppr encoder attached to the roller. Data from the different sensors are obtained at a frequency of 1 kHz [28].

### Experimental design

A cross-over study was designed to examine the influence of the body position adopted during upper-body dynamic resistance training on IOP values. The 10-RM loads during the bench press exercise performed in supine and seated positions were determined in the first testing session. In the second session, participants randomly performed 6 exercise sets (3 exercise intensities (control, medium intensity, and high intensity) × 2 body positions (supine and seated)) and the IOP was measured before exercise, after each of the 10 repetitions, and after 10 s of recovery (12 IOP measurements for each experimental condition). The six exercise sets were performed in a randomized order in order to minimize the possible effects of fatigue on the findings of the present study, and environmental conditions were kept constant during the course of the experiment (∼ 22 °C and ∼ 60% humidity).

### Statistical analyses

Firstly, the normal distribution of the data (Shapiro–Wilk test) and the homogeneity of variances (Levene’s test) were checked. Also, a two-way repeated measures ANOVA (2 × 3: body position (supine and seated) and exercise intensity (control, medium intensity, and high intensity)) was performed to assess if baseline IOP measurements were comparable.

For the main analysis, we performed a repeated measures ANOVA for IOP (2 × 3 × 12), considering the body position (supine and seated), exercise intensity (control, medium intensity, and high intensity), and point of measure (baseline, repetitions 1–10, and recovery (a total of 12 measurements)) as within-participant factors. Multiple comparisons were performed, and they were corrected with the Holm–Bonferroni procedure. The magnitude of the changes was reported as Cohen’s *d* effect size (*d*) and partial eta squared (*η*_p_^2^) for *T* and *F* tests, respectively. An alpha value of 0.05 was considered as statistically significant. The JASP statistics package (version 0.16.2) was used for statistical analyses.

## Results

The normal distribution of the data and the homogeneity of variances were confirmed (all *p*-values > 0.05). The analysis of baseline IOP values showed statistically significant differences for the main factor “body position” (*F*_1,22_ = 47.85, *p* < 0.001, *η*_p_^2^ = 0.69), with greater values in the supine than in the seated position. However, no differences were reached for the main factor “exercise intensity” or the interaction “body position × exercise intensity” (*F*_2,44_ = 2.67, *p* = 0.081; *F*_2,44_ = 1.38, *p* = 0.262; respectively).

**Fig. 2:**
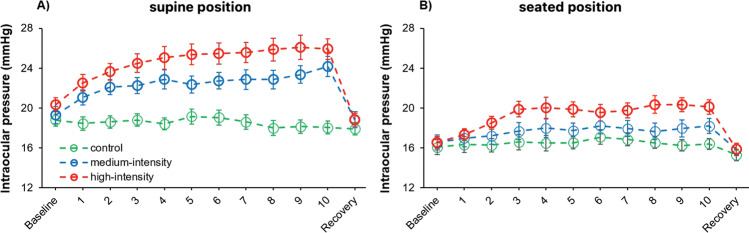
Intraocular pressure behavior in supine (panel **A**) and seated (panel **B**) positions for the control, medium-intensity, and high-intensity conditions. Error bars show the standard error

**Fig. 3:**
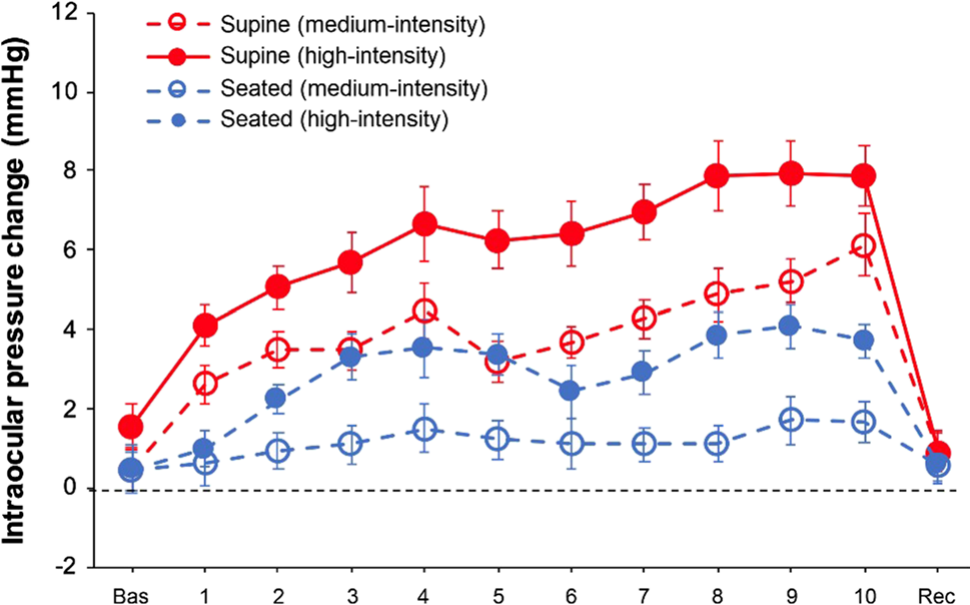
Changes in intraocular pressure levels between the medium- and high-intensity conditions with respect to the control condition during the bench press performed in supine and seated positions. Error bars show the standard error of the difference

The analysis of IOP showed statistically significant differences for the main effects of “body position” (*F*_1,22_ = 107.50, *p* < 0.001, *η*_p_^2^ = 0.83), “exercise intensity” (*F*_2,44_ = 85.29, *p* < 0.001, *η*_p_^2^ = 0.80), and “point of measure” (*F*_2,44_ = 38.34, *p* < 0.001, *η*_p_^2^ = 0.64). Also, there were statistically significant differences for the interactions “body position × exercise intensity” (*F*_2,44_ = 29.44, *p* < 0.001, *η*_p_^2^ = 0.57), “body position × point of measure” (*F*_11,242_ = 8.45, *p* = 0.004, *η*_p_^2^ = 0.11), “exercise intensity × point of measure” (*F*_22,484_ = 13.48, < 0.001, *η*_p_^2^ = 0.38), and “body position × exercise intensity × point of measure” (*F*_22,484_ = 2.34, < 0.001, *η*_p_^2^ = 0.10) (Fig. 2). Post hoc analyses revealed greater IOP values in the high-intensity condition in comparison to the medium-intensity and control conditions (corrected *p*-value < 0.001, Cohen’s *d* = 1.17; and corrected *p*-value < 0.001, Cohen’s *d* = 2.72, respectively), as well as for the medium-intensity condition when compared to the control condition (corrected *p*-value < 0.001, Cohen’s *d* = 1.54). Complementarily, we calculated the IOP change associated with exercise performance in supine and seated positions (Fig. 3).

## Discussion

This study was designed to examine the influence of the body position adopted during the execution of the bench press exercise on IOP. Our data revealed a mediating effect of the body position on IOP behavior, obtaining greater IOP increments when the bench press was performed in the supine compared to the seated position. Additionally, and in agreement with previous studies, (i) IOP increased progressively over the course of the set [22], (ii) IOP increments were positively associated with exercise intensity [13, 14], and (iii) IOP returned to baseline values immediately after the cessation of the effort [29, 30]. Based on the current results, performing the dynamic bench press to or near to muscular failure, and especially while adopting a supine position, should be discouraged in individuals who need to minimize IOP increments. These findings may have important applications for exercise prescription in glaucoma patients or those at risk.

This is the first study that has explored during virtually the same exercise, bilateral upper-body push, the effect of the body position adopted during the execution of the exercise on IOP levels. Our preliminary hypothesis was confirmed because the supine bench press exercise caused greater IOP increments than the seated bench press exercise. As displayed in Fig. 2, the IOP peaks in the supine and seated positions were of approximately 26 and 20 mmHg, respectively, which evidence the possible clinical relevance of these differences. To our knowledge, this is the first investigation assessing the impact of the body position adopted during resistance training on IOP, although these results agree with previous studies using non-physical activities [16–21]. In line with previous studies [19–21], the baseline IOP differences between the seated and supine positions ranged from 1 to 3 mmHg. However, during the execution of the bench press exercise, the IOP differences between the supine and seated positions are greater during the physical effort in comparison to baseline conditions (average difference during 10 repetitions of ~ 5.5 mmHg vs. ~ 3 mmHg in baseline conditions). Based on the present outcomes, it is reasonable to prioritize the adoption of standing/seated positions instead of lying down for the execution of the bench press exercise when maintaining stable IOP levels is desired or necessary [31, 32].

Our data corroborate previous findings about the factors mediating the IOP response to resistance training. First, the execution of highly demanding physical effort is known to provoke an acute and transient IOP rise, which these changes being positively associated with the accumulated level of effort [22, 33]. In the current study, there was a linear IOP rise during exercise performance in the medium- and high-intensity conditions and while adopting both the seated and supine positions (coefficients of correlation (Pearson’s *r*) ranging from 0.75 to 0.93). Second, greater intensities (i.e., loads lifted) in resistance training are linked to more abrupt IOP increments [13–15, 22, 34–37], which is also observed in the present study. Lastly, these IOP effects have demonstrated to be transient, namely, IOP values return to baseline levels after a few seconds of exercise cessation [14, 29, 30, 33]. Here, we found that 10 s of passive recovery were enough to reach baseline IOP levels. This set of results supports that those studies designed to capture IOP changes as a function of exercise with a post-exercise measurement may underestimate the impact of exercise on IOP levels.

The physiological mechanisms responsible for the mediating effects of body position on the IOP response to resistance training cannot be ruled out from this investigation; however, there are several plausible explanations to these findings. The execution of highly demanding resistance training is accompanied by the Valsalva maneuver, which permits to enhance trunk stability [38]. Nevertheless, the hemodynamic changes linked to the Valsalva maneuver are also dependent on the body position adopted, with these differences being attributed to differences in intrathoracic blood volume and venous return [39]. In addition, the adoption of recumbent positions has been associated with an increased episcleral venous pressure and ocular tissues congestion, including the choroid (i.e., the most vascularized ocular tissue) [16, 18, 40]. All these factors may have contributed to the differences in the IOP response to dynamic resistance training in the seated and supine positions.

### Limitations and future research

The findings of this study reveal that performing 10 repetitions of the bench press exercise leading to muscular failure caused greater IOP rises when performed in supine compared to seated position. Nevertheless, there are different factors that may limit the validity of these results, and they must be listed. First, several factors such as individuals’ fitness level [34, 41–43], type of exercise [30, 36], or participants’ sex [14, 44] have demonstrated to influence the IOP response to exercise, and thus, the results of this study should be interpreted with caution in this regard. Second, the experimental sample was formed by healthy young adults, and the physical capacity and physiological responsiveness to exercise in other populations (e.g., elderly subjects with chronic conditions) may be different [45]. Third, ocular hemodynamics in glaucoma patients after postural change is known to be altered [46], which support that further studies are needed to determine the IOP responsiveness to resistance training while adopting different body positions in glaucoma patients. Lastly, this work is limited to the acute effects of resistance training on IOP, and future studies should be designed to determine the long-term effects of exercise practice on glaucoma prevention and management.

## Conclusions

The differences in IOP values between the supine and seated positions are accentuated during effort. There was a progressive IOP rise in both body positions, exercise intensity was positively associated with the magnitude of the IOP increment, and IOP returned to baseline levels immediately after exercise cessation. This set of findings support previous evidence on the IOP response to highly demanding resistance training, and incorporate novel insights into the mediating role of the body position adopted during the execution of resistance training exercises on IOP levels. Our results suggest that the adoption of a seated/standing position (instead of a supine/prone position) should be prioritized for maintaining more stable IOP levels, and may help to the management and prevention of glaucoma. The external validity of these results for glaucoma patients needs to be tested in future studies.

## References

[1] Pedersen BK, Saltin B (2015). Exercise as medicine - evidence for prescribing exercise as therapy in 26 different chronic diseases. Scand J Med Sci Sport.

[2] Warburton DER, Bredin SSD (2016). Reflections on physical activity and health: what should we recommend?. Can J Cardiol.

[3] Thornton JS, Frémont P, Khan K (2016). Physical activity prescription: a critical opportunity to address a modifiable risk factor for the prevention and management of chronic disease: a position statement by the Canadian Academy of Sport and Exercise Medicine. Br J Sports Med.

[4] Tham YC, Li X, Wong TY (2014). Global prevalence of glaucoma and projections of glaucoma burden through 2040: a systematic review and meta-analysis. Ophthalmology.

[5] Reis TF, Paula JS, Furtado JM (2022). Primary glaucomas in adults: epidemiology and public health-a review. Clin Exp Ophthalmol.

[6] Leske MC, Heijl A, Hussein M, Bengtsson B, Hyman LKE (2003). Factors for glaucoma progression and the effect of treatment: the Early Manifest Glaucoma Trial. Arch Ophthalmol.

[7] Caprioli J, Coleman AL (2008). Intraocular pressure fluctuation: a risk factor for visual field progression at low intraocular pressures in the Advanced Glaucoma Intervention Study. Ophthalmology.

[8] De MoraesJuthani CGVVJ, Liebmann JM (2011). Risk factors for visual field progression in treated glaucoma. Arch Ophthalmol.

[9] Asrani S, Zeimer R, Wilensky J (2000). Large diurnal fluctuations in intraocular pressure are an independent risk factor in patients with glaucoma. J Glaucoma.

[10] Zhu MM, Lai JSM, Choy BNK (2018). Physical exercise and glaucoma: a review on the roles of physical exercise on intraocular pressure control, ocular blood flow regulation, neuroprotection and glaucoma-related mental health. Acta Ophthalmol.

[11] Janicijevic D, Redondo B, Jiménez R (2021). Intraocular pressure responses to walking with surgical and FFP2/N95 face masks in primary open-angle glaucoma patients. Graefe’s Arch Clin Exp Ophthalmol.

[12] Najmanova E, Pluhacek F, Botek M (2016). Intraocular pressure response to moderate exercise during 30-min recovery. Optom Vis Sci.

[13] Vera J, García-Ramos A, Jiménez R, Cárdenas D (2017). The acute effect of strength exercises at different intensities on intraocular pressure. Graefe’s Arch Clin Exp Ophthalmol.

[14] Vera J, Raimundo J, García-Durán B, Pérez-Castilla A, Redondo B, Delgado G, ... García-Ramos A (2019) Acute intraocular pressure changes during isometric exercise and recovery: the influence of exercise type and intensity, and participant´s sex. J Sports Sci 37(19):2213–2219 10.1080/02640414.2019.1626072 31177968

[15] Vaghefi E, Shon C, Reading S (2021). Intraocular pressure fluctuation during resistance exercise. BMJ Open Ophthalmol.

[16] Prata TS, De MoraesKanadani CGVFN (2010). Posture-induced intraocular pressure changes: considerations regarding body position in glaucoma patients. Surv Ophthalmol.

[17] Turner DC, Samuels BC, Huisingh C (2017). The magnitude and time course of IOP change in response to body position change in nonhuman primates measured using continuous IOP telemetry. Investig Ophthalmol Vis Sci.

[18] Malihi M, Sit AJ (2012). Effect of head and body position on intraocular pressure. Ophthalmology.

[19] Sobczak M, Asejczyk M, Geniusz M (2022). Does body position, age, and heart rate induce IOP’s changes?. Eur J Ophthalmol.

[20] Najmanova E, Pluhacek F, Haklova M (2019) Intraocular pressure response affected by changing of sitting and supine positions. Acta Ophthalmol 98(3):e368–e372 10.1111/aos.14267 PMC7216979 31602816

[21] Vera J, Redondo B, Molina R (2020). Acute intraocular pressure responses to reading: the influence of body position. J Glaucoma.

[22] Vera J, Jiménez R, Redondo B (2019). Effect of the level of effort during resistance training on intraocular pressure. Eur J Sport Sci.

[23] Kang H (2021) Sample size determination and power analysis using the G* Power software. J Educ Eval Health Prof 18 10.3352/jeehp.2021.18.17 PMC8441096 34325496

[24] Faul F, Erdfelder E, Lang A-G, Buchner A (2007). G*Power 3: a flexible statistical power analysis program for the social, behavioral, and biomedical sciences. Behav Res Methods.

[25] Badakere SV, Chary R, Choudhari NS (2021). Agreement of intraocular pressure measurement of Icare ic200 with Goldmann applanation tonometer in adult eyes with normal cornea. Ophthalmol Glaucoma.

[26] Nakakura S, Asaoka R, Terao E (2021). Evaluation of rebound tonometer iCare IC200 as compared with IcarePRO and Goldmann applanation tonometer in patients with glaucoma. Eye Vis.

[27] Rodriguez-Perea Á, Jerez-Mayorga D, García-Ramos A (2021). Reliability and concurrent validity of a functional electromechanical dynamometer device for the assessment of movement velocity. Proc Inst Mech Eng Part P J Sport Eng Technol.

[28] Sánchez-Sánchez AJ, Chirosa-Ríos LJ, Chirosa-Ríos IJ (2021). Test-retest reliability of a functional electromechanical dynamometer on swing eccentric hamstring exercise measures in soccer players. PeerJ.

[29] Vera J, Jiménez R, Redondo B (2019). Investigating the immediate and cumulative effects of isometric squat exercise for different weight loads on intraocular pressure: a pilot study. Sports Health.

[30] Vera J, Redondo B, Koulieris GA (2020). Intraocular pressure responses to four different isometric exercises in men and women. Optom Vis Sci.

[31] Coleman AL, Miglior S (2008). Risk factors for glaucoma onset and progression. Surv Ophthalmol.

[32] De Moraes CG, Mansouri K, Liebmann JM, Ritch R (2018). Association between 24-hour intraocular pressure monitored with contact lens sensor and visual field progression in older adults with glaucoma. JAMA Ophthalmol.

[33] Bakke EF, Hisdal J, Semb SO (2009). Intraocular pressure increases in parallel with systemic blood pressure during isometric exercise. Investig Ophthalmol Vis Sci.

[34] Vera J, Jiménez R, Redondo B, Cárdenas D, De Moraes CG, Garcia-Ramos A (2017) Intraocular pressure responses to maximal cycling sprints against different resistances: the influence of fitness level. J Glaucoma 26(10):881–887 10.1097/IJG.0000000000000749 28834828

[35] Vera J, Redondo B, Molina R (2019). Influence of holding weights of different magnitudes on intraocular pressure and anterior eye biometrics. Graefe’s Arch Clin Exp Ophthalmol.

[36] Rüfer F, Schiller J, Klettner A (2014). Comparison of the influence of aerobic and resistance exercise of the upper and lower limb on intraocular pressure. Acta Ophthalmol.

[37] Vera J, Jiménez R, Redondo B (2020). Impact of resistance training sets performed until muscular failure with different loads on intraocular pressure and ocular perfusion pressure. Eur J Ophthalmol.

[38] Hackett DA, Chow C-M (2013). The Valsalva maneuver: its effect on intra-abdominal pressure and safety issues during resistance exercise. J Strength Cond Res.

[39] Pstras L, Thomaseth K, Waniewski J (2016). The Valsalva manoeuvre: physiology and clinical examples. Acta Physiol.

[40] Friberg TR, Sanborn GWR (1987). Intraocular and episcleral venous pressure increase during inverted posture. Am J Ophthalmol.

[41] Vera J, Jiménez R, Redondo B (2019). Effect of a maximal treadmill test on intraocular pressure and ocular perfusion pressure: the mediating role of fitness level. Eur J Ophthalmol.

[42] Janicijevic D, Redondo B, Jiménez R (2022). The intraocular pressure lowering-effect of low-intensity aerobic exercise is greater in fitter individuals: a cluster analysis. Res Sport Med.

[43] Vera J, Jiménez R, Redondo B (2018). Fitness level modulates intraocular pressure responses to strength exercises. Curr Eye Res.

[44] Pérez-Castilla A, García-Ramos A, Redondo B, Andrés FR, Jiménez R, Vera J (2020) Determinant factors of intraocular pressure responses to a maximal isometric handgrip test: hand dominance, handgrip strength and sex. Curr Eye Res 46(1):64–70 10.1080/02713683.2020.1780265 32511035

[45] Hoffmann TC, Maher CG, Briffa T (2016). Prescribing exercise interventions for patients with chronic conditions. CMAJ.

[46] Galambos P, Vafiadis J, Vilchez SE (2006). Compromised autoregulatory control of ocular hemodynamics in glaucoma patients after postural change. Ophthalmology.

